# Bloodstream Infections among HIV-Infected Outpatients, Southeast Asia

**DOI:** 10.3201/eid1610.091686

**Published:** 2010-10

**Authors:** Jay K. Varma, Kimberly D. McCarthy, Theerawit Tasaneeyapan, Patama Monkongdee, Michael E. Kimerling, Eng Buntheoun, Delphine Sculier, Chantary Keo, Praphan Phanuphak, Nipat Teeratakulpisarn, Nibondh Udomsantisuk, Nguyen H. Dung, Nguyen T.N. Lan, Nguyen T.B. Yen, Kevin P. Cain

**Affiliations:** Author affiliations: Thailand Ministry of Public Health–US Centers for Disease Control and Prevention Collaboration, Nonthaburi, Thailand (J.K. Varma, T. Tasaneeyapan, P. Monkongdee);; Centers for Disease Control and Prevention, Atlanta, Georgia, USA (J.K. Varma, K.D. McCarthy, K.P. Cain);; Bill and Melinda Gates Foundation, Seattle, Washington, USA (M.E. Kimerling);; Centers for Disease Control and Prevention, Phnom Penh, Cambodia (E. Buntheoun);; Sihanouk Hospital Center of Hope, Phnom Penh, (D. Sculier);; Institute of Tropical Medicine, Antwerp, Belgium (D. Sculier);; Institute Pasteur Cambodia, Phnom Penh (C. Keo); Thai Red Cross AIDS Research Center, Bangkok, Thailand (P. Phanuphak, N. Teeratakulpisarn);; Chulalongkorn University, Bangkok (N. Udomsantisuk);; Pham Ngoc Thach Hospital for Tuberculosis and Lung Diseases, Ho Chi Minh City, Vietnam (N.H. Dung, N.T.N. Lan, N.T.B. Yen)

**Keywords:** Tuberculosis and other mycobacteria, HIV/AIDS and other retroviruses, diagnosis, bacteria, Salmonella, cryptoccoci, penicilliosis, fungi, research

## MEDSCAPE CME

Medscape, LLC is pleased to provide online continuing medical education (CME) for this journal article, allowing clinicians the opportunity to earn CME credit. This activity has been planned and implemented in accordance with the Essential Areas and policies of the Accreditation Council for Continuing Medical Education through the joint sponsorship of Medscape, LLC and Emerging Infectious Diseases. Medscape, LLC is accredited by the ACCME to provide continuing medical education for physicians. Medscape, LLC designates this educational activity for a maximum of 0.5 *AMA PRA Category 1 Credits*™. Physicians should only claim credit commensurate with the extent of their participation in the activity. All other clinicians completing this activity will be issued a certificate of participation. To participate in this journal CME activity: (1) review the learning objectives and author disclosures; (2) study the education content; (3) take the post-test and/or complete the evaluation at www.medscapecme.com/journal/eid; (4) view/print certificate.

## Learning Objectives

Upon completion of this activity, participants will be able to:

- Describe overall prevalence of bloodstream infections (BSIs) and prevalence of specific BSIs in HIV-infected outpatients, based on a southeast Asian study sample.
- Describe risk factors for overall and specific BSI in HIV-infected persons in that sample.

## MEDSCAPE CME EDITOR

**Karen L. Foster, MA**, Technical Writer/Editor, *Emerging Infectious Diseases. Disclosure: Karen L. Foster, MA,*** ***has disclosed no relevant financial relationships.*

## MEDSCAPE CME AUTHOR

**Laurie Barclay, MD**, freelance writer and reviewer, Medscape, LLC. *Disclosure: Laurie Barclay, MD, has disclosed no relevant financial relationships.*

## AUTHORS

*Disclosure:* ***Jay K. Varma, MD; Kimberly D. McCarthy, MS; Theerawit Tasaneeyapan, MSc; Patama Monkongdee, MSc; Michael Kimerling, MD, MPH; Eng Buntheoun, MD; Delphine Sculier, MD, MSc; Chantary Keo; Praphan Phanuphak, MD, PhD; Nipat Teeratakulpisarn, MD; Nibondh Udomsantisuk, MD; Nguyen H. Dung, MD, MS; Nguyen T.N. Lan, MD, PhD; Nguyen T.B. Yen, MD;*** *and* ***Kevin P. Cain, MD,*** *have disclosed no relevant financial relationships.*

## References

[1] Archibald LK, Dulk WO, Pallangyo KJ, Reller LB. Fatal *Mycobacterium tuberculosis* bloodstream infections in febrile hospitalized adults in Dar es Salaam, Tanzania. Clin Infect Dis. 1998;26:290–6. 10.1086/516297 9502444

[2] Peters RP, Zijlstra EE, Schijffelen MJ, Walsh AL, Joaki G, Kumwenda JJ, A prospective study of bloodstream infections as cause of fever in Malawi: clinical predictors and implications for management. Trop Med Int Health. 2004;9:928–34. 10.1111/j.1365-3156.2004.01288.x 15304000

[3] Ssali FN, Kamya MR, Wabwire-Mangen F, Kasasa S, Joloba M, Williams D, A prospective study of community-acquired bloodstream infections among febrile adults admitted to Mulago Hospital in Kampala, Uganda. J Acquir Immune Defic Syndr Hum Retrovirol. 1998;19:484–9. 10.1097/00042560-199812150-00007 9859962

[4] Oplustil CP, Leite OH, Oliveira MS, Sinto SI, Uip DE, Boulos M, Detection of mycobacteria in the bloodstream of patients with acquired immunodeficiency syndrome in a university hospital in Brazil. Braz J Infect Dis. 2001;5:252–9. 10.1590/S1413-86702001000500003 11779451

[5] Ramachandran R, Swaminathan S, Somasundaram S, Asgar VN, Paramesh P, Paramasivan CN. Mycobacteremia in tuberculosis patients with HIV infection. Indian J Tuberc. 2002;50:29–31.

[6] McDonald LC, Archibald LK, Rheanpumikankit S, Tansuphaswadikul S, Eampokalap B, Nwanyanawu O, Unrecognized *Mycobacterium tuberculosis* bacteremia among hospital inpatients in less developed countries. Lancet. 1999;354:1159–63. 10.1016/S0140-6736(98)12325-5 10513709

[7] Archibald LK, McDonald LC, Rheanpumikankit S, Tansuphaswadikul S, Chaovanich A, Eampokalap B, Fever and human immunodeficiency virus infection as sentinels for emerging mycobacterial and fungal bloodstream infections in hospitalized patients >15 years old, Bangkok. J Infect Dis. 1999;180:87–92. 10.1086/314836 10353865

[8] Talbot EA, Hay Burgess DC, Hone NM, Iademarco MF, Mwasekaga MJ, Moffat HJ, Tuberculosis serodiagnosis in a predominantly HIV-infected population of hospitalized patients with cough, Botswana, 2002. Clin Infect Dis. 2004;39:e1–7. 10.1086/421388 15206074

[9] von Reyn CF. The significance of bacteremic tuberculosis among persons with HIV infection in developing countries. AIDS. 1999;13:2193–5. 10.1097/00002030-199911120-00001 10563704

[10] Archibald LK, McDonald LC, Nwanyanwu O, Kazembe P, Dobbie H, Tokars J, A hospital-based prevalence survey of bloodstream infections in febrile patients in Malawi: implications for diagnosis and therapy. J Infect Dis. 2000;181:1414–20. 10.1086/315367 10762572

[11] Bell M, Archibald LK, Nwanyanwu O, Dobbie H, Tokars J, Kazembe PN, Seasonal variation in the etiology of bloodstream infections in a febrile inpatient population in a developing country. Int J Infect Dis. 2001;5:63–9. 10.1016/S1201-9712(01)90027-X 11468099

[12] Gordon MA, Walsh AL, Chaponda M, Soko D, Mbvwinji M, Molyneux ME, Bacteraemia and mortality among adult medical admissions in Malawi—predominance of non-Typhi salmonellae and *Streptococcus pneumoniae.* J Infect. 2001;42:44–9. 10.1053/jinf.2000.0779 11243753

[13] Arthur G, Nduba VN, Kariuki SM, Kimari J, Bhatt SM, Gilks CF. Trends in bloodstream infections among human immunodeficiency virus–infected adults admitted to a hospital in Nairobi, Kenya, during the last decade. Clin Infect Dis. 2001;33:248–56. 10.1086/321820 11418886

[14] Peters RP, Zijlstra EE, Schijffelen MJ, Walsh AL, Joaki G, Kumwenda JJ, A prospective study of bloodstream infections as cause of fever in Malawi: clinical predictors and implications for management. Trop Med Int Health. 2004;9:928–34. 10.1111/j.1365-3156.2004.01288.x 15304000

[15] Gilks CF, Brindle RJ, Otieno LS, Simani PM, Newnham RS, Bhatt SM, Life-threatening bacteraemia in HIV-1 seropositive adults admitted to hospital in Nairobi, Kenya. Lancet. 1990;336:545–9. 10.1016/0140-6736(90)92096-Z 1975046

[16] Vugia DJ, Kiehlbauch JA, Yeboue K, N'Gbichi JM, Lacina D, Maran M, Pathogens and predictors of fatal septicemia associated with human immunodeficiency virus infection in Ivory Coast, West Africa. J Infect Dis. 1993;168:564–70. 10.1093/infdis/168.3.564 8394859

[17] Gordon MA, Walsh AL, Chaponda M, Soko D, Mbvwinji M, Molyneux EM, Bacteraemia and mortality among adult medical admissions in Malawi—predominance of non-Typhi salmonellae and *Streptococcus pneumoniae.* J Infect. 2001;42:44–9. 10.1053/jinf.2000.0779 11243753

[18] Joint United Nations Program on HIV/AIDS. 2008 Report on the global HIV/AIDS epidemic [cited 2010 Aug 4]. http://www.unaids.org/en/KnowledgeCentre/HIVData/GlobalReport/2008/2008_Global_report.asp

[19] Phetsouvanh R, Phongmany S, Soukaloun D, Rasachak B, Soukhaseum V, Frichithavong K, Causes of community-acquired bacteremia and patterns of antimicrobial resistance in Vientiane, Laos. Am J Trop Med Hyg. 2006;75:978–85. 17124000 PMC2213713

[20] Chierakul W, Rajanuwong A, Wuthiekanun V, Teerawattanasook N, Gasiprong M, Simpson A, The changing pattern of bloodstream infections associated with the rise in HIV prevalence in northeastern Thailand. Trans R Soc Trop Med Hyg. 2004;98:678–86. 15363648 10.1016/j.trstmh.2004.01.011

[21] Hoa NT, Diep TS, Wain J, Parry CM, Hien TT, Smith MD, Community-acquired septicaemia in southern Viet Nam: the importance of multidrug-resistant *Salmonella* Typhi. Trans R Soc Trop Med Hyg. 1998;92:503–8. 10.1016/S0035-9203(98)90891-4 9861362

[22] Monkongdee P, McCarthy KD, Cain KP, Tasaneeyapan T, Nguyen HD, Nguyen TN, Yield of acid-fast smear and mycobacterial culture for tuberculosis diagnosis in people with HIV. Am J Respir Crit Care Med. 2009;180:903–8. 10.1164/rccm.200905-0692OC 19628775

[23] Butler WR, Guthertz LS. Mycolic acid analysis by high-performance liquid chromatography for identification of *Mycobacterium* species. Clin Microbiol Rev. 2001;14:704–26. 10.1128/CMR.14.4.704-726.2001 11585782 PMC88994

[24] Weinstein MP. Blood culture contamination: persisting problems and partial progress. J Clin Microbiol. 2003;41:2275–8. 10.1128/JCM.41.6.2275-2278.2003 12791835 PMC156489

[25] Weinstein MP, Towns ML, Quartey SM, Mirrett S, Reimer LG, Parmigiani G, The clinical significance of positive blood cultures in the 1990s: a prospective comprehensive evaluation of the microbiology, epidemiology, and outcome of bacteremia and fungemia in adults. Clin Infect Dis. 1997;24:584–602. 9145732 10.1093/clind/24.4.584

[26] Tumbarello M, Tacconelli E, Donati KG, Citton R, Leone F, Spanu T, HIV-associated bacteremia: how it has changed in the highly active antiretroviral therapy (HAART) era. J Acquir Immune Defic Syndr. 2000;23:145–51. 10737429 10.1097/00126334-200002010-00006

[27] Meynard JL, Guiguet M, Fonquernie L, Lefebvre B, Lalande V, Honore I, Impact of highly active antiretroviral therapy on the occurrence of bacteraemia in HIV-infected patients and their epidemiologic characteristics. HIV Med. 2003;4:127–32. 10.1046/j.1468-1293.2003.00146.x 12702133

[28] Ortega M, Almela M, Soriano A, Marco F, Martinez JA, Munoz A, Bloodstream infections among human immunodeficiency virus-infected adult patients: epidemiology and risk factors for mortality. Eur J Clin Microbiol Infect Dis. 2008;27:969–76. 10.1007/s10096-008-0531-5 18449581

[29] Shah S, Demissie M, Lambert L, Ahmed J, Leulseged S, Kebede T, Intensified tuberculosis case finding among HIV-infected persons from a voluntary counseling and testing center in Addis Ababa, Ethiopia. J Acquir Immune Defic Syndr. 2009;50:537–45. 10.1097/QAI.0b013e318196761c 19223783

[30] Chheng P, Tamhane A, Natpratan C, Tan V, Lay V, Sar B, Pulmonary tuberculosis among clients visiting a voluntary confidential counseling and testing center, Cambodia. Int J Tuberc Lung Dis. 2008;12(Suppl 1):54–62. 18302824

[31] Wood R, Maartens G, Lombard CJ. Risk factors for developing tuberculosis in HIV-1–infected adults from communities with a low or very high incidence of tuberculosis. J Acquir Immune Defic Syndr. 2000;23:75–80. 10708059 10.1097/00126334-200001010-00010

[32] World Health Organization. Global tuberculosis control—epidemiology, strategy, financing. Geneva: The Organization; 2009 [cited 2010 Aug 4]. http://www.who.int/tb/publications/global_report/2009/en/index.html

[33] World Health Organization. Interim policy on collaborative TB/HIV activities. Geneva: The Organization; 2004 [cited 2010 Aug 4]. http://www.who.int/hiv/pub/tb/tbhiv/en/

[34] Chuck SL, Sande MA. Infections with *Cryptococcus neoformans* in the acquired immunodeficiency syndrome. N Engl J Med. 1989;321:794–9. 10.1056/NEJM198909213211205 2671735

[35] Duong TA. Infection due to *Penicillium marneffei*, an emerging pathogen: review of 155 reported cases. Clin Infect Dis. 1996;23:125–30. 10.1093/clinids/23.1.125 8816141

[36] Gordon MA, Banda HT, Gondwe M, Gordon SB, Boeree MJ, Walsh AL, Non-typhoidal salmonella bacteraemia among HIV-infected Malawian adults: high mortality and frequent recrudescence. AIDS. 2002;16:1633–41. 10.1097/00002030-200208160-00009 12172085

[37] Lamy B, Roy P, Carret G, Flandrois JP, Delignette-Muller ML. What is the relevance of obtaining multiple blood samples for culture? A comprehensive model to optimize the strategy for diagnosing bacteremia. Clin Infect Dis. 2002;35:842–50. 10.1086/342383 12228821

[38] Baggett HC, Peruski LF, Olsen SJ, Thamthitiwat S, Rhodes J, Dejsirilert S, Incidence of pneumococcal bacteremia requiring hospitalization in rural Thailand. Clin Infect Dis. 2009;48:S65–74. 10.1086/596484 19191621

