# Supplementary material for: Bloodstream Infections among HIV-Infected Outpatients, Southeast Asia
Source: Emerg Infect Dis. 2010 Oct;16(10):1569–75. doi: 10.3201/eid1610.091686 (PMC3294385; doi:10.3201/eid1610.091686)
Supplement: Related texts

# Research article: Bloodstream Infections among HIV-Infected Outpatients, Southeast Asia

**DOI:** 10.3201/eid1610.091686

**Authors:** Jay K. Varma, Kimberly D. McCarthy, Theerawit Tasaneeyapan, Patama Monkongdee, Michael E. Kimerling, Eng Buntheoun, Delphine Sculier, Chantary Keo, Praphan Phanuphak, Nipat Teeratakulpisarn, Nibondh Udomsantisuk, Nguyen H. Dung, Nguyen T.N. Lan, Nguyen T.B. Yen, Kevin P. Cain

**Affiliations:** Author affiliations: Thailand Ministry of Public Health–US Centers for Disease Control and Prevention Collaboration, Nonthaburi, Thailand (J.K. Varma, T. Tasaneeyapan, P. Monkongdee);; Centers for Disease Control and Prevention, Atlanta, Georgia, USA (J.K. Varma, K.D. McCarthy, K.P. Cain);; Bill and Melinda Gates Foundation, Seattle, Washington, USA (M.E. Kimerling);; Centers for Disease Control and Prevention, Phnom Penh, Cambodia (E. Buntheoun);; Sihanouk Hospital Center of Hope, Phnom Penh, (D. Sculier);; Institute of Tropical Medicine, Antwerp, Belgium (D. Sculier);; Institute Pasteur Cambodia, Phnom Penh (C. Keo); Thai Red Cross AIDS Research Center, Bangkok, Thailand (P. Phanuphak, N. Teeratakulpisarn);; Chulalongkorn University, Bangkok (N. Udomsantisuk);; Pham Ngoc Thach Hospital for Tuberculosis and Lung Diseases, Ho Chi Minh City, Vietnam (N.H. Dung, N.T.N. Lan, N.T.B. Yen)

## Abstract

Bloodstream infections (BSIs) are a major cause of illness in HIV-infected persons. To evaluate prevalence of and risk factors for BSIs in 2,009 HIV-infected outpatients in Cambodia, Thailand, and Vietnam, we performed a single Myco/F Lytic blood culture. Fifty-eight (2.9%) had a clinically significant BSI (i.e., a blood culture positive for an organism known to be a pathogen). *Mycobacterium tuberculosis* accounted for 31 (54%) of all BSIs, followed by fungi (13 [22%]) and bacteria (9 [16%]). Of patients for whom data were recorded about antiretroviral therapy, 0 of 119 who had received antiretroviral therapy for >14 days had a BSI, compared with 3% of 1,801 patients who had not. In multivariate analysis, factors consistently associated with BSI were fever, low CD4+ T-lymphocyte count, abnormalities on chest radiograph, and signs or symptoms of abdominal illness. For HIV-infected outpatients with these risk factors, clinicians should place their highest priority on diagnosing tuberculosis.

Bloodstream infections (BSIs) are a major cause of illness in HIV-infected persons. A series of studies, most of which were conducted in sub-Saharan Africa during the 1990s, demonstrated a high prevalence of BSIs (ranging from 10% to 63%) among hospitalized HIV-infected persons who had fever (*1**–**17*). In studies that measured clinical outcomes, the in-hospital death rate for patients with a BSI was high (19%–47%). A variety of pathogens cause BSIs in febrile, hospitalized persons with HIV, most notably non-Typhi *Salmonella* spp. (6%–15%) and *Mycobacterium tuberculosis* (2%–19%). BSI with *M. tuberculosis* appears to be particularly lethal, causing death during hospitalization in up to 47% of patients (*9*). Although untreated BSIs are believed to lead rapidly to severe illness, sepsis, and death, patients with BSIs may be able to be identified before they are ill enough to require hospitalization, potentially improving clinical outcomes. Despite the large number of studies that have evaluated BSIs in HIV-infected persons, all previous studies have focused on patients seeking care at hospitals because of fever and did not evaluate infections among outpatients with or without fever.

Although overall transmission rates have declined and antiretroviral therapy (ART) has become more widely available, HIV infection remains a major public health problem in Southeast Asia (*18*). Previous studies of BSI in Southeast Asia enrolled only inpatients, and only 1 evaluated a predominantly HIV-infected population (*1**,**19**–**21*). In this study, we prospectively enrolled patients from multiple HIV testing and treatment clinics in Cambodia, Thailand, and Vietnam to assess BSI prevalence, etiology, and risk factors in outpatients with HIV.

## Methods

### Enrollment and Specimen Collection

From September 2006 through July 2008, HIV-infected persons were enrolled consecutively from community outpatient facilities that perform HIV counseling, testing, and clinical care: 4 clinics in Cambodia (2 in Băntéay Méan Cheăy Province, 1 in Bătdâmbâng Province, 1 in Phnom Penh); 1 in Bangkok, Thailand; and 3 in Ho Chi Minh City, Vietnam. At each facility, HIV-infected persons who came to the clinic during the enrollment period were screened for eligibility and, if eligible, were offered enrollment. The enrolled group comprised both persons newly diagnosed with HIV and persons previously diagnosed with HIV, some of whom were receiving ART. Patients were asked to participate in the study regardless of the presence or absence of symptoms or prior suspicion of clinical illness. Patients were eligible for the study if they had documented HIV infection and were >6 years of age. Because the study was designed primarily to evaluate different strategies for diagnosing tuberculosis (TB) in HIV-infected persons, patients were excluded if they had undergone TB screening with chest radiograph or sputum smears in the previous 3 months and if they had taken medications with anti-TB activity within the past month (*22*).

After providing written informed consent, patients underwent a standardized interview and physical examination, chest radiograph, and blood testing for complete blood cell count and CD4+ T lymphocytes. Patients were asked about a broad range of symptoms and exposures during the past 4 weeks. Trained phlebotomists obtained 5 mL of blood and directly injected it into Myco/F Lytic bottles (Becton Dickinson, Franklin Lakes, NJ, USA). Bottles were kept at room temperature and shielded from light, then transferred to a centralized laboratory, where they were placed into an automated blood culture instrument (BACTEC 9050/9120/9240 system; Becton Dickinson).

The human subjects review committees at Centers for Disease Control and Prevention (Atlanta, GA, USA) approved the study. Collaborating institutions in each country also approved it.

### Specimen Processing

A detailed description of processing and testing of the nonsputum specimens has been published (*22*). Myco/F Lytic bottles were incubated for 42 days in a BACTEC 9050/9120/9240 instrument. Cultures flagged as positive by the instruments were removed, acid-fast bacilli (AFB) smears were carried out, and specimens were subcultured onto blood agar plates. AFB-positive cultures were subcultured onto 2 Lowenstein-Jensen media slants. AFB-negative Myco/F Lytic cultures that had growth on blood agar plates underwent bacterial or fungal identification. Blood cultures <42 days old with no organisms found on smear and blood agar plates were returned to the instrument. All Myco/F Lytic cultures were removed after 42 days, visually inspected for growth, subcultured onto 2 Lowenstein-Jensen slants, incubated for an additional 3 weeks, and then discarded. Cultures positive for *M. tuberculosis* were identified by using the niacin production and nitrate reduction tests. Nontuberculous mycobacteria (NTM) were speciated by using high-performance liquid chromatography or the Genotype Mycobacterium CM/AS **assay (Hain** Lifescience, Nehren, Germany) **(***23***).**

### Data Analysis

Blood cultures were classified as negative, positive for a likely contaminant, or positive for a clinically significant pathogen. Because only 1 blood specimen for culture was drawn from each patient, the identity of the organism was used as the only criteria for determining whether the blood culture result was due to contamination or a clinically significant pathogen (*24**,**25*).

We calculated proportions and medians to describe patient characteristics and calculated bivariate odds ratios (ORs) and 95% confidence intervals (CIs) to analyze factors associated with clinically significant BSI. To calculate adjusted ORs for clinically significant BSI, factors significant in bivariate analysis at p<0.05 and factors hypothesized a priori to be associated with BSI were entered in a multiple logistic regression model, and a final model was chosen through stepwise automated variable selection. We assessed all factors for colinearity; for colinear factors, we retained the factor that had largest OR and that we judged to be most clinically meaningful. For the bivariate analysis, we analyzed only observations with complete (non-missing) data; for the multivariate analysis, we created a separate missing values stratum for 3 variables (CD4 cell count, hemoglobin level, leukocyte count) to maximize the number of observations in the final model. Analyses were conducted by the same approach to evaluate independent clinical predictors of mycobacterial, bacterial, and fungal BSI. All analyses were conducted in SAS version 9.1 (SAS Institute, Cary, NC, USA).

## Results

### Enrollment

Of 2,115 patients evaluated, 2,013 (95.2%) were eligible for the study; of eligible patients, 2,009 were enrolled. Reasons for ineligibility included current or recent TB treatment (87 patients), use of medications with anti-TB activity in the past month (5), recent TB screening (3), age <7 years (2), and other or missing reasons for noneligibility (5).

### Patient Characteristics

Almost half (945 [47.0%]) of the patients enrolled were from Cambodia. Median age was 31 years (interquartile range [IQR] 27–38); 1,019 (50.7%) patients were male. A clinically significant BSI was found in 58 (2.9%) patients and a contaminant in 131 (6.5%). *M. tuberculosis* caused 31 (54%) BSIs; 13 (22%) BSIs were caused by fungi, 9 (16%) by bacteria, and 5 (9%) by NTM (Table 1).

**Table 1:** Clinically significant organisms isolated from cultures of blood samples from 2,009 HIV-infected outpatients from Thailand, Cambodia, and Vietnam, September 2006–July 2008

Organism	No. (%), n = 58
Mycobacteria	36 (62)
* Mycobacterium tuberculosis*	31 (86)
* M. avium-intracellulare*	3 (8)
* M. simiae*	1 (3)
Non-tuberculous mycobacteria not identified	1 (3)
Other bacteria	9 (16)
Non-Typhi *Salmonella* spp.	2 (22)
* S. cholerasius*	3 (33)
*Pseudomonas* spp.	1 (11)
*Neisseria* spp.	2 (22)
* Escherichia coli*	1 (11)
Fungi	13 (22)
* Cryptococcus neoformans*	6 (46)
* Penicillium marneffei*	5 (38)
* Histoplasma capsulatum*	1 (8)
Non-albicans *Candida* spp.	1 (8)

### Risk Factors for BSI

In bivariate analysis, a large number of symptoms, signs, chest radiography findings, and laboratory studies were associated with BSI (Table A1). BSI was associated with recent diagnosis of HIV infection, which we defined as receipt of an HIV diagnosis within the 14 days before enrollment, to account for delays between initial diagnosis and visit to an HIV clinic (OR 1.80, 95% CI 1.06–3.05). The 1 sign or symptom most strongly associated with BSI was temperature >38^◦^C, which was documented in 25 (43%) patients with BSI compared with 5% of those without BSI (OR 13.58, 95% CI 7.78–23.68). CD4 cell count was strongly associated with BSI. The median CD4 count for patients with a BSI was 15 cells/mm^3^ (IQR 8–50), compared with 261 cells/mm^3^ (IQR 102–405) for those without a BSI (p<0.01). Of patients with BSI, 50 (86%) had CD4 count <100 cells/mm^3^, including 44 (76%) with a CD4 count <50 cells/mm^3^. Only 5 (9%) patients with BSI had CD4 count >200 cells/mm^3^. Of enrolled patients, 83 (4%) had a temperature >38°C and a CD4 count <100 cells/mm^3^. Of these, 21 (25%) had a BSI, including 14 caused by mycobacteria (13 *M. tuberculosis* and 1 NTM) and 7 caused by fungi. The characteristic that was most protective was receipt of ART. Of patients for whom data were recorded about receipt of ART, 0 of 119 who had received ART for at least 14 days had a BSI, compared with 3% of 1,801 who did not receive ART or who had started ART <14 days previously (OR 0).

In multivariate analysis, several factors were significantly associated with BSI. These factors were loss of appetite, nausea or vomiting; temperature >38°C, oral hairy leukoplakia, CD4 count <100 cells/mm^3^, anemia, leukocytosis, and paratracheal adenopathy or a miliary pattern on a chest radiograph (Table 2).

**Table 2:** Multivariate analysis of risk factors for clinically significant BSI caused by mycobacterial or other bacterial infection in HIV-infected outpatients, Thailand, Cambodia, and Vietnam, September 2006–July 2008*

Characteristic	Adjusted odds ratio (95% CI) for BSI		
	Any pathogen†	Mycobacteria‡	Bacteria§
CD4 cell count <100/mm^3^	5.8 (2.5–13.7)	11.2 (3.0–41.3)	–
Female sex	0.4 (0.2–0.9)	–	–
Fever in past 24 h	–	–	4.7 (1.2–17.6)
Loss of appetite in past 24 h	2.0 (1.0–3.9)	–	–
Nausea or vomiting in past 24 h	2.5 (1.2–5.0)	–	–
Shaking chills in past 24 h	–	3.1 (1.1–9.1)	–
Difficulty breathing	–	4.1 (1.5–11.1)	–
Diarrhea in past 24 h	–	4.2 (1.6–11.3)	–
Jaundice	–	–	12.5 (1.4–112.3)
Ever injected drug	–	–	–
Temperature >38°C	3.2 (1.6–6.3)	5.7 (2.1–15.4)	–
Heart rate >100 bpm	–	–	–
Oral hairy leukoplakia	2.8 (1.4–5.7)	–	–
Hemoglobin level <12 g/dL	4.8 (2.0–11.7)	–	–
Leukocyte count >12 × 10^3^/µL	3.7 (1.4–9.3)	15.3 (4.9–48.2)	–
Thrombocyte count <100,000 cells/µL	–	7.0 (1.8–27.6)	–
Paratracheal adenopathy on chest radiograph	5.0 (2.1–11.6)	11.3 (3.7–34.3)	–
Miliary pattern on chest radiograph	6.0 (1.8–19.5)	14.2 (3.4–58.6)	–
Took antimicrobial medication other than cotrimoxazole	–	5.1 (1.6–16.8)	–

*BSI, bloodstream infection; CI, confidence interval; bpm, beats per minute; –, variable was not included in the model. †1,961 patients in final model. ‡1,944 patients in final model. §1,961 patients in final model.

### Risk Factors for Mycobacterial, Fungal, and Bacterial BSIs

When we restricted the multivariate analysis to different subsets of BSI, we found that several factors were independently associated with mycobacterial BSI: shaking chills, difficulty breathing, diarrhea, temperature >38°C, leukocytosis, thrombocytopenia, paratracheal adenopathy or miliary pattern on the chest radiograph, and receipt of an antibiotic drug other than co-trimoxazole (Table 2). The only independent predictors of bacterial BSI were jaundice and self-report of fever in the previous 24 hours. No factors were statistically significant in the analysis of fungal BSI.

## Discussion

In this large study of HIV-infected persons in Southeast Asia, 1 in 35 outpatients had a BSI; the highest prevalence was in patients with low CD4 counts and clinical signs of infection. *M. tuberculosis* remains one of the most common causes of BSI in HIV-infected persons who live in resource-limited settings. In all analyses performed, several factors were consistently associated with BSI: self-reported fever or documented elevated temperature, low CD4 count, abnormalities on chest radiograph, and signs or symptoms of abdominal illness.

BSI correlated strongly with immunosuppression. In fact, 10% of outpatients with HIV and CD4 count <100 cells/mm^3^ had a BSI, a prevalence similar to that seen in a previous study of febrile, HIV-infected inpatients in Thailand (*6*). No patients who received ART had a BSI, consistent with the observation from other settings that highly active ART may reduce the incidence of bacteremia in HIV-infected persons (*26**–**28*). *M. tuberculosis* was the most frequent pathogen isolated in our study, findings consistent with studies that have shown that undiagnosed TB disease is common in patients with newly diagnosed HIVinfection and that the invasiveness of TB increases with declining CD4 cell counts and with the absence of ART (*29**–**31*). These findings are particularly valid in countries with a high incidence of TB, such as Thailand, Cambodia, and Vietnam; in these 3 countries, the estimated incidence is >140 TB cases (all forms) per 100,000 persons (*32*). We found that strong predictors of *M. tuberculosis* BSI included clinical features that suggest pulmonary TB, including difficulty breathing and adenopathy or a miliary pattern shown on a chest radiograph. In an analysis published separately, we demonstrated that the incremental yield of blood culture for detecting TB was extremely low in HIV-infected persons who have 3 sputum specimens cultured on liquid media (*22*). Our study, therefore, further supports the World Health Organization policy of focusing on pulmonary, rather than extrapulmonary, TB case finding and of recommending routine, regular TB screening for HIV-infected patients (*33*).

More than one fifth of all BSIs were attributable to fungi, but we were unable to identify any clinical characteristics independently associated with fungal BSI. Because cryptococcosis and penicilliosis are commonly associated with advanced immunosuppression and have high death rates if left untreated, further studies are needed to improve case finding for and prevention of these infections (*34**,**35*). In contrast, we found that self-reported fever and the finding of jaundice on physical examination were strong predictors of bacterial infection. The reasons for an association with jaundice are unclear, but the association is consistent with our finding that most bacterial infections were of enteric origin. Symptoms of abdominal illness, such as loss of appetite and nausea or vomiting, also were associated with BSI caused by any pathogen. Our finding that non-Typhi *Salmonella* spp. infections were the most common bacterial infection in HIV patients is consistent with results of other studies and provides further evidence that efforts are needed to prevent invasive salmonellosis in HIV-infected persons, through improvements in food and water safety and the development of new vaccines (*36*).

Our study has several limitations. First, we collected only 1 blood culture per patient and used only 1 type of culture media, which potentially reduced the sensitivity for detection of bacteremia (*37*). This limitation is a likely explanation for the lack of *Streptococcus pneumoniae* detected in our study. Invasive pneumococcal disease is a common cause of bacteremia in HIV-infected patients throughout the world, but *S. pneumoniae* is challenging to isolate from blood. In addition, 12% of study patients were receiving co-trimoxazole preventive therapy, and our study was conducted among outpatients, a population less likely to have undiagnosed severe disease caused by a virulent pathogen, such as pneumococcus. Although other investigators in Southeast Asia have found similarly low pneumococcal isolation rates and have speculated that this is attributable to low incidence, at least 1 high-quality study demonstrated that the incidence of invasive pneumococcal disease in Thailand is similar to that in other regions (*19**–**21**,**38*).

A major strength of our study, however, is that, unlike all previous studies, which were conducted at single referral hospitals, our study was conducted at multiple urban and rural clinical facilities in 3 countries. Thus, our results can be broadly generalized to HIV-infected patients throughout Southeast Asia.

Mycobacterial, fungal, and bacterial BSIs remain a major health problem for HIV-infected persons in Southeast Asia. Any HIV-infected outpatients (regardless of whether they have newly diagnosed HIV, are newly seeking care, or are already receiving care) who report experiencing fever or abdominal symptoms in the previous day, have a temperature >38^◦^C or jaundice on physical examination, or have a chest radiograph demonstrating paratracheal adenopathy or a miliary pattern, have a high likelihood of a BSI, particularly if their CD4 count is <100 cells/mm^3^. In such patients, blood culture, when available, should be performed immediately to facilitate diagnosis and accelerate access to treatment of BSI. Regardless of blood culture availability, clinicians should place their highest priority on early diagnosis and treatment of pulmonary TB. Ultimately, increasing use of ART most likely will have the greatest effect on reducing BSIs.

## Appendix

**Table A1:** Characteristics of 2,009 HIV-infected outpatients and associated BSI, Thailand, Cambodia, and Vietnam, September 2006–July 2008*

Characteristic	All, no. (%), N = 2,009	Have BSI†, no./total (%), n = 58	Do not have BSI, no./total (%), n = 1,951	OR (95% CI)
Demographics				
Country of origin				
Thailand	630 (31)	5/58 (9)	625/1,951 (32)	Referent
Cambodia	945 (47)	31/58 (53)	914/1,951 (47)	4.2 (1.6–11.0)
Vietnam	434 (22)	22/58 (38)	412/1,951 (21)	6.7 (2.5–17.8)
Median age, y (IQR)‡	31 (27–38)	32 (27–36)	31 (27–38)	NS
Male sex	1,019 (51)	40/58 (69)	979/1,951 (50)	2.21 (1.26–3.88)
Medical history				
History of injection drug use	255 (13)	15/58 (26)	240/1,950 (12)	2.49 (1.36–4.54)
HIV diagnosed in past 14 d§	853 (43)	33/58 (57)	820/1,951 (42)	1.80 (1.06–3.05)
Received ART for >14 d	119 (6)	0/58 (0)	119/1,862 (6)	0
Took co-trimoxazole	232 (12)	12/58 (21)	220/1,950 (11)	2.05 (1.07–3.93)
Took other antimicrobial drug	194 (10)	12/58 (21)	182/1,951 (9)	2.54 (1.32–4.87)
Constitutional symptoms¶				
Fever	398 (20)	36/58 (62)	362/1,950 (19)	10.54 (4.51–24.65)
Fever lasting >14 d	313 (16)	32/58 (55)	281/1,948 (14)	7.30 (4.29–12.44)
Shaking chills	204 (10)	19/58 (33)	185/1,946 (10)	4.64 (2.63–8.19)
Night sweats	309 (15)	25/58 (43)	284/1,949 (15)	4.44 (2.60–7.58)
Night sweats lasting >14 d	199 (10)	16/57 (28)	183/1,943 (9)	3.75 (2.07–6.82)
Respiratory symptoms				
Cough	688 (34)	39/58 (67)	649/1,949 (33)	4.11 (2.36–7.17)
Every day	811 (40)	40/58 (69)	771/1,949 (40)	3.40 (1.93–5.97)
Recently worsened	72 (4)	7/58 (12)	65/1947 (3)	3.97 (1.74–9.09)
Lasting >14 d	380 (19)	22/58 (38)	358/1,946 (18)	2.71 (1.58–4.66)
Difficulty breathing	428 (21)	32/58 (55)	396/1,951 (20)	4.83 (2.85–8.2)
Chest pain	419 (21)	30/58 (52)	389/1,951 (20)	4.30 (2.54–7.29)
Gastrointestinal symptoms				
Mouth or throat pain	403 (20)	27/58 (47)	376/1,951 (19)	3.65 (2.15–6.19)
Pain with swallowing	282 (14)	21/58 (36)	261/1,949 (13)	3.67 (2.12–6.37)
Abdominal pain	321 (16)	20/58 (35)	301/1,951 (15)	2.89 (1.66–5.03)
Loss of appetite	471 (23)	38/57 (67)	433/1,951 (22)	7.01 (4.00–12.29)
Nausea or vomiting	250 (12)	22/58 (38)	228/1,950 (12)	4.62 (2.67–7.98)
Diarrhea	185 (9)	19/58 (33)	166/1,949 (9)	5.23 (2.96–9.26)
Diarrhea lasting >14 d	94 (5)	7/58 (12)	87/1,948 (5)	2.94 (1.29–6.66)
Neurologic-muscular symptoms				
Lightheadedness	507 (25)	28/58 (48)	479/1,947 (25)	2.86 (1.69–4.84)
Fatigue	875 (44)	48/58 (83)	827/1,949 (42)	6.51 (3.28–12.95)
Muscle weakness	523 (26)	33/58 (57)	490/1,950 (25)	3.93 (2.32–6.68)
Difficulty sleeping	630 (31)	36/58 (62)	594/1,948 (31)	3.73 (2.18–6.39)
Numbness in feet	420 (21)	25/58 (43)	395/1,949 (20)	2.98 (1.75–5.07)
Signs				
Temperature >38^◦^C	128 (6)	25/58 (43)	103/1,949 (5)	13.58 (7.78–23.68)
Heart rate >100 beats/min	231 (11)	22/58 (38)	209/19,48 (11)	5.08 (2.94–8.81)
Body mass index <18.5	546 (27)	34/58 (59)	512/1,950 (26)	3.98 (2.34–6.77)
Thrush	249 (12)	25/58 (43)	224/1,949 (12)	5.83 (3.41–9.99)
Oral hairy leukoplakia	166 (8)	19/58 (33)	147/1,949 (8)	5.97 (3.37–10.6)
Oral ulcerations	128 (6)	12/58 (21)	116/1,948 (6)	4.12 (2.12–7.99)
Jaundice	15 (1)	3/58 (5)	12/1,949 (1)	8.80 (2.42–32.09)
Any lymphadenopathy#	238 (12)	18/58 (31)	220/1,945 (11)	3.53 (1.99–6.26)
Head and neck lymphadenopathy	181 (9)	18/58 (31)	163/1,945 (8)	4.92 (2.76–8.78)
Papular rash	402 (20)	18/58 (31)	384/1,949 (20)	1.83 (1.04–3.23)
Needle tracks on arms	42 (2)	5/58 (9)	37/1,949 (2)	4.88 (1.84–12.9)
CD4+ T-lymphocyte count				
CD4 count, median (IQR)	252 (92–401)	15 (8–50)	261 (102–405)	p < 0.01
<50 cells/mm^3^	350 (17)	44/58 (76)	306/1,934 (16)	16.72 (9.05–30.89)
<100 cells/mm^3^	529 (26)	50/58 (86)	479/1,934 (25)	18.98 (8.94–40.33)
<200 cells/mm^3^	810 (40)	53/58 (91)	757/1,934 (39)	16.48 (6.56–41.42)
<250 cells/mm^3^	988 (49)	53/58 (91)	935/1,934 (48)	11.33 (4.51–28.46)
Karnofsky score <70	85 (4)	7/58 (12)	78/1,945 (4)	3.29 (1.44–7.47)
Hematologic tests				
Hemoglobin level <12 g/dL	777 (39)	46/53 (87)	731/1,913 (38)	10.63 (4.77–23.66)
Leukocyte count >12 × 10^3^ cells/µL	1,189 (59)	12/53 (23)	50/1,893 (3)	10.79 (5.35–21.77)
Thrombocyte count <100 × 10^3^cells/µL	145 (7)	11/58 (19)	134/1,951 (7)	3.17 (1.61–6.26)
Chest radiograph				
Abnormal chest radiograph	457 (23)	36/58 (62)	421/1,925 (22)	5.85 (3.4–10.04)
Interstitial pattern	82 (4)	10/58 (17)	72/1,951 (4)	5.44 (2.64–11.18)
Any infiltrate	266 (13)	19/58 (33)	247/1,951 (13)	3.36 (1.91–5.91)
Miliary pattern	25 (1)	7/58 (12)	18/1,951 (1)	14.74 (5.90–36.85)
Pleural effusion	39 (2)	4/58 (7)	35/1,951 (2)	4.06 (1.39–11.81)
Mass or nodule	57 (3)	8/58 (14)	49/1,951 (3)	6.21 (2.80–13.8)
Paratracheal adenopathy	70 (3)	14/58 (24)	56/1,951 (3)	10.77 (5.58–20.78)
>1/3 of either lung abnormal	108 (5)	14/58 (24)	94/1,951 (5)	6.29 (3.33–11.87)
Any lower lung field abnormality	194 (10)	22/58 (38)	172/1,951 (9)	6.32 (3.64–10.99)
Any upper lung field abnormality	258 (13)	20/58 (35)	238/1,951 (12)	3.79 (2.17–6.62)

*Characteristics displayed include those postulated to be associated with BSI that were statistically significant in bivariate analysis. BSI, bloodstream infection; OR, odds ratio; CI, confidence interval; IQR, interquartile range; NS, not significant; ART, antiretroviral therapy. †Patients whose blood culture grew a contaminant or had no growth were classified as having no BSI. ‡Age was analyzed as a continuous variable with an OR calculated by using logistic regression; p = 0.46. §The exact date of HIV diagnosis was missing for 12 patients. ¶Unless specified, patients were asked about any occurrence of this symptom in the past 24 h. #Defined as having a peripheral, noninguinal lymph node >1 cm in diameter or a peripheral, inguinal lymph node >2 cm in diameter.

## References

[1] Archibald LK, Dulk WO, Pallangyo KJ, Reller LB. Fatal *Mycobacterium tuberculosis* bloodstream infections in febrile hospitalized adults in Dar es Salaam, Tanzania. Clin Infect Dis. 1998;26:290–6. 10.1086/5162979502444

[2] Peters RP, Zijlstra EE, Schijffelen MJ, Walsh AL, Joaki G, Kumwenda JJ, A prospective study of bloodstream infections as cause of fever in Malawi: clinical predictors and implications for management. Trop Med Int Health. 2004;9:928–34. 10.1111/j.1365-3156.2004.01288.x15304000

[3] Ssali FN, Kamya MR, Wabwire-Mangen F, Kasasa S, Joloba M, Williams D, A prospective study of community-acquired bloodstream infections among febrile adults admitted to Mulago Hospital in Kampala, Uganda. J Acquir Immune Defic Syndr Hum Retrovirol. 1998;19:484–9. 10.1097/00042560-199812150-000079859962

[4] Oplustil CP, Leite OH, Oliveira MS, Sinto SI, Uip DE, Boulos M, Detection of mycobacteria in the bloodstream of patients with acquired immunodeficiency syndrome in a university hospital in Brazil. Braz J Infect Dis. 2001;5:252–9. 10.1590/S1413-8670200100050000311779451

[5] Ramachandran R, Swaminathan S, Somasundaram S, Asgar VN, Paramesh P, Paramasivan CN. Mycobacteremia in tuberculosis patients with HIV infection. Indian J Tuberc. 2002;50:29–31.

[6] McDonald LC, Archibald LK, Rheanpumikankit S, Tansuphaswadikul S, Eampokalap B, Nwanyanawu O, Unrecognized *Mycobacterium tuberculosis* bacteremia among hospital inpatients in less developed countries. Lancet. 1999;354:1159–63. 10.1016/S0140-6736(98)12325-510513709

[7] Archibald LK, McDonald LC, Rheanpumikankit S, Tansuphaswadikul S, Chaovanich A, Eampokalap B, Fever and human immunodeficiency virus infection as sentinels for emerging mycobacterial and fungal bloodstream infections in hospitalized patients >15 years old, Bangkok. J Infect Dis. 1999;180:87–92. 10.1086/31483610353865

[8] Talbot EA, Hay Burgess DC, Hone NM, Iademarco MF, Mwasekaga MJ, Moffat HJ, Tuberculosis serodiagnosis in a predominantly HIV-infected population of hospitalized patients with cough, Botswana, 2002. Clin Infect Dis. 2004;39:e1–7. 10.1086/42138815206074

[9] von Reyn CF. The significance of bacteremic tuberculosis among persons with HIV infection in developing countries. AIDS. 1999;13:2193–5. 10.1097/00002030-199911120-0000110563704

[10] Archibald LK, McDonald LC, Nwanyanwu O, Kazembe P, Dobbie H, Tokars J, A hospital-based prevalence survey of bloodstream infections in febrile patients in Malawi: implications for diagnosis and therapy. J Infect Dis. 2000;181:1414–20. 10.1086/31536710762572

[11] Bell M, Archibald LK, Nwanyanwu O, Dobbie H, Tokars J, Kazembe PN, Seasonal variation in the etiology of bloodstream infections in a febrile inpatient population in a developing country. Int J Infect Dis. 2001;5:63–9. 10.1016/S1201-9712(01)90027-X11468099

[12] Gordon MA, Walsh AL, Chaponda M, Soko D, Mbvwinji M, Molyneux ME, Bacteraemia and mortality among adult medical admissions in Malawi—predominance of non-Typhi salmonellae and *Streptococcus pneumoniae.* J Infect. 2001;42:44–9. 10.1053/jinf.2000.077911243753

[13] Arthur G, Nduba VN, Kariuki SM, Kimari J, Bhatt SM, Gilks CF. Trends in bloodstream infections among human immunodeficiency virus–infected adults admitted to a hospital in Nairobi, Kenya, during the last decade. Clin Infect Dis. 2001;33:248–56. 10.1086/32182011418886

[14] Peters RP, Zijlstra EE, Schijffelen MJ, Walsh AL, Joaki G, Kumwenda JJ, A prospective study of bloodstream infections as cause of fever in Malawi: clinical predictors and implications for management. Trop Med Int Health. 2004;9:928–34. 10.1111/j.1365-3156.2004.01288.x15304000

[15] Gilks CF, Brindle RJ, Otieno LS, Simani PM, Newnham RS, Bhatt SM, Life-threatening bacteraemia in HIV-1 seropositive adults admitted to hospital in Nairobi, Kenya. Lancet. 1990;336:545–9. 10.1016/0140-6736(90)92096-Z1975046

[16] Vugia DJ, Kiehlbauch JA, Yeboue K, N'Gbichi JM, Lacina D, Maran M, Pathogens and predictors of fatal septicemia associated with human immunodeficiency virus infection in Ivory Coast, West Africa. J Infect Dis. 1993;168:564–70. 10.1093/infdis/168.3.5648394859

[17] Gordon MA, Walsh AL, Chaponda M, Soko D, Mbvwinji M, Molyneux EM, Bacteraemia and mortality among adult medical admissions in Malawi—predominance of non-Typhi salmonellae and *Streptococcus pneumoniae.* J Infect. 2001;42:44–9. 10.1053/jinf.2000.077911243753

[18] Joint United Nations Program on HIV/AIDS. 2008 Report on the global HIV/AIDS epidemic [cited 2010 Aug 4]. http://www.unaids.org/en/KnowledgeCentre/HIVData/GlobalReport/2008/2008_Global_report.asp

[19] Phetsouvanh R, Phongmany S, Soukaloun D, Rasachak B, Soukhaseum V, Frichithavong K, Causes of community-acquired bacteremia and patterns of antimicrobial resistance in Vientiane, Laos. Am J Trop Med Hyg. 2006;75:978–85.17124000PMC2213713

[20] Chierakul W, Rajanuwong A, Wuthiekanun V, Teerawattanasook N, Gasiprong M, Simpson A, The changing pattern of bloodstream infections associated with the rise in HIV prevalence in northeastern Thailand. Trans R Soc Trop Med Hyg. 2004;98:678–86.1536364810.1016/j.trstmh.2004.01.011

[21] Hoa NT, Diep TS, Wain J, Parry CM, Hien TT, Smith MD, Community-acquired septicaemia in southern Viet Nam: the importance of multidrug-resistant *Salmonella* Typhi. Trans R Soc Trop Med Hyg. 1998;92:503–8. 10.1016/S0035-9203(98)90891-49861362

[22] Monkongdee P, McCarthy KD, Cain KP, Tasaneeyapan T, Nguyen HD, Nguyen TN, Yield of acid-fast smear and mycobacterial culture for tuberculosis diagnosis in people with HIV. Am J Respir Crit Care Med. 2009;180:903–8. 10.1164/rccm.200905-0692OC19628775

[23] Butler WR, Guthertz LS. Mycolic acid analysis by high-performance liquid chromatography for identification of *Mycobacterium* species. Clin Microbiol Rev. 2001;14:704–26. 10.1128/CMR.14.4.704-726.200111585782PMC88994

[24] Weinstein MP. Blood culture contamination: persisting problems and partial progress. J Clin Microbiol. 2003;41:2275–8. 10.1128/JCM.41.6.2275-2278.200312791835PMC156489

[25] Weinstein MP, Towns ML, Quartey SM, Mirrett S, Reimer LG, Parmigiani G, The clinical significance of positive blood cultures in the 1990s: a prospective comprehensive evaluation of the microbiology, epidemiology, and outcome of bacteremia and fungemia in adults. Clin Infect Dis. 1997;24:584–602.914573210.1093/clind/24.4.584

[26] Tumbarello M, Tacconelli E, Donati KG, Citton R, Leone F, Spanu T, HIV-associated bacteremia: how it has changed in the highly active antiretroviral therapy (HAART) era. J Acquir Immune Defic Syndr. 2000;23:145–51.1073742910.1097/00126334-200002010-00006

[27] Meynard JL, Guiguet M, Fonquernie L, Lefebvre B, Lalande V, Honore I, Impact of highly active antiretroviral therapy on the occurrence of bacteraemia in HIV-infected patients and their epidemiologic characteristics. HIV Med. 2003;4:127–32. 10.1046/j.1468-1293.2003.00146.x12702133

[28] Ortega M, Almela M, Soriano A, Marco F, Martinez JA, Munoz A, Bloodstream infections among human immunodeficiency virus-infected adult patients: epidemiology and risk factors for mortality. Eur J Clin Microbiol Infect Dis. 2008;27:969–76. 10.1007/s10096-008-0531-518449581

[29] Shah S, Demissie M, Lambert L, Ahmed J, Leulseged S, Kebede T, Intensified tuberculosis case finding among HIV-infected persons from a voluntary counseling and testing center in Addis Ababa, Ethiopia. J Acquir Immune Defic Syndr. 2009;50:537–45. 10.1097/QAI.0b013e318196761c19223783

[30] Chheng P, Tamhane A, Natpratan C, Tan V, Lay V, Sar B, Pulmonary tuberculosis among clients visiting a voluntary confidential counseling and testing center, Cambodia. Int J Tuberc Lung Dis. 2008;12(Suppl 1):54–62.18302824

[31] Wood R, Maartens G, Lombard CJ. Risk factors for developing tuberculosis in HIV-1–infected adults from communities with a low or very high incidence of tuberculosis. J Acquir Immune Defic Syndr. 2000;23:75–80.1070805910.1097/00126334-200001010-00010

[32] World Health Organization. Global tuberculosis control—epidemiology, strategy, financing. Geneva: The Organization; 2009 [cited 2010 Aug 4]. http://www.who.int/tb/publications/global_report/2009/en/index.html

[33] World Health Organization. Interim policy on collaborative TB/HIV activities. Geneva: The Organization; 2004 [cited 2010 Aug 4]. http://www.who.int/hiv/pub/tb/tbhiv/en/

[34] Chuck SL, Sande MA. Infections with *Cryptococcus neoformans* in the acquired immunodeficiency syndrome. N Engl J Med. 1989;321:794–9. 10.1056/NEJM1989092132112052671735

[35] Duong TA. Infection due to *Penicillium marneffei*, an emerging pathogen: review of 155 reported cases. Clin Infect Dis. 1996;23:125–30. 10.1093/clinids/23.1.1258816141

[36] Gordon MA, Banda HT, Gondwe M, Gordon SB, Boeree MJ, Walsh AL, Non-typhoidal salmonella bacteraemia among HIV-infected Malawian adults: high mortality and frequent recrudescence. AIDS. 2002;16:1633–41. 10.1097/00002030-200208160-0000912172085

[37] Lamy B, Roy P, Carret G, Flandrois JP, Delignette-Muller ML. What is the relevance of obtaining multiple blood samples for culture? A comprehensive model to optimize the strategy for diagnosing bacteremia. Clin Infect Dis. 2002;35:842–50. 10.1086/34238312228821

[38] Baggett HC, Peruski LF, Olsen SJ, Thamthitiwat S, Rhodes J, Dejsirilert S, Incidence of pneumococcal bacteremia requiring hospitalization in rural Thailand. Clin Infect Dis. 2009;48:S65–74. 10.1086/59648419191621

# Research article: Bloodstream Infections among HIV-Infected Outpatients, Southeast Asia

**DOI:** 10.3201/eid1610.091686

**Authors:** Jay K. Varma, Kimberly D. McCarthy, Theerawit Tasaneeyapan, Patama Monkongdee, Michael E. Kimerling, Eng Buntheoun, Delphine Sculier, Chantary Keo, Praphan Phanuphak, Nipat Teeratakulpisarn, Nibondh Udomsantisuk, Nguyen H. Dung, Nguyen T.N. Lan, Nguyen T.B. Yen, Kevin P. Cain

**Affiliations:** Author affiliations: Thailand Ministry of Public Health–US Centers for Disease Control and Prevention Collaboration, Nonthaburi, Thailand (J.K. Varma, T. Tasaneeyapan, P. Monkongdee);; Centers for Disease Control and Prevention, Atlanta, Georgia, USA (J.K. Varma, K.D. McCarthy, K.P. Cain);; Bill and Melinda Gates Foundation, Seattle, Washington, USA (M.E. Kimerling);; Centers for Disease Control and Prevention, Phnom Penh, Cambodia (E. Buntheoun);; Sihanouk Hospital Center of Hope, Phnom Penh, (D. Sculier);; Institute of Tropical Medicine, Antwerp, Belgium (D. Sculier);; Institute Pasteur Cambodia, Phnom Penh (C. Keo); Thai Red Cross AIDS Research Center, Bangkok, Thailand (P. Phanuphak, N. Teeratakulpisarn);; Chulalongkorn University, Bangkok (N. Udomsantisuk);; Pham Ngoc Thach Hospital for Tuberculosis and Lung Diseases, Ho Chi Minh City, Vietnam (N.H. Dung, N.T.N. Lan, N.T.B. Yen)

## Earning CME Credit

To obtain credit, you should first read the journal article. After reading the article, you should be able to answer the following, related, multiple-choice questions. To complete the questions and earn continuing medical education (CME) credit, please go to **www.medscapecme.com/journal/eid**. Credit cannot be obtained for tests completed on paper, although you may use the worksheet below to keep a record of your answers. You must be a registered user on Medscape.com. If you are not registered on Medscape.com, please click on the New Users: Free Registration link on the left hand side of the website to register. Only one answer is correct for each question. Once you successfully answer all post-test questions you will be able to view and/or print your certificate. For questions regarding the content of this activity, contact the accredited provider, CME@medscape.net. For technical assistance, contact CME@webmd.net. American Medical Association’s Physician’s Recognition Award (AMA PRA) credits are accepted in the US as evidence of participation in CME activities. For further information on this award, please refer to http://www.ama-assn.org/ama/pub/category/2922.html. The AMA has determined that physicians not licensed in the US who participate in this CME activity are eligible for *AMA PRA Category 1 Credits*™. Through agreements that the AMA has made with agencies in some countries, AMA PRA credit is acceptable as evidence of participation in CME activities. If you are not licensed in the US and want to obtain an AMA PRA CME credit, please complete the questions online, print the certificate and present it to your national medical association.

## Article Title: Bloodstream Infections among HIV-Infected Outpatients, Southeast Asia

## CME Questions

**1. Based on the above southeast Asia study by Dr Varma and colleagues, which of the following statements about prevalence of overall bloodstream infections (BSIs) and of specific BSIs in HIV-infected persons is correct?**A. BSIs are commonly encountered in HIV-infected outpatients, noted in up to 5%B. The most common bacterial infection was *Streptococcus pneumoniae*C. *Mycobacterium tuberculosis* was the most common pathogen responsible for BSID. *M. tuberculosis* accounted for approximately one quarter of all BSI**2. Your patient is a 23-year-old Cambodian male recently diagnosed with HIV infection. Based on the above study, which of the following is most likely to be associated with increased risk for overall BSI?**A. CD4 = 150 cells/mm^3^B. Vomiting and abdominal tendernessC. Generalized rashD. Subnormal temperature**3. Based on the above study, which of the following statements regarding increased risk for specific BSI is most likely correct for the patient in question 2?**A. Hilar adenopathy on chest x-ray suggests tuberculosis infectionB. Difficulty breathing suggests fungal BSIC. Jaundice was not a significant predictor of any class of pathogenD. Loss of appetite was a specific predictor of bacterial infection

## Activity Evaluation

**Table:** 

1. The activity supported the learning objectives.				
Strongly Disagree				Strongly Agree
1	2	3	4	5
2. The material was organized clearly for learning to occur.				
Strongly Disagree				Strongly Agree
1	2	3	4	5
3. The content learned from this activity will impact my practice.				
Strongly Disagree				Strongly Agree
1	2	3	4	5
4. The activity was presented objectively and free of commercial bias.				
Strongly Disagree				Strongly Agree
1	2	3	4	5
